# A Very "Moderate" Policy

**Published:** 1912-02-24

**Authors:** 


### The Hospital

The Workers' Newspaper of Administrative Medicine and Institutional Life, Administration, National Insurance and Health.

No. 1337, Vol. LI. SATURDAY, FEBRUARY 24th, 1912. PRICE ONE PENNY.

### A VERY "MODERATE" POLICY.

On February 8 a new development of the situation as between the medical profession and the Insurance Act was provided by a manifesto which is headed "An Appeal for a Strong but Moderate Policy." Now moderation is one of the cardinal virtues, and in everyday affairs is perhaps fully as useful and valuable as strength. Indeed the truest strength is that which knows itself secure enough to practise moderation. Yet the perusal of the document referred to leaves the impression that the policy advocated is moderate much more by virtue of its weakness than its strength; and further, that it is calculated to divide and disunite the medical profession just as its new-found solidarity is beginning to make an impression.

The manifesto is signed by about fifty practitioners from various parts of England who are "committed by no pledges to support or oppose the present Council of the British Medical Association." Perhaps not; yet the confidence which this statement might otherwise arouse is somewhat mitigated by the inclusion among the signatures of men who have publicly defended the action of the Council in its dealings with the Government before the Bill became an Act, and by the fact that all but about half a dozen of them are actually officials of the Association.

The next assertion that challenges criticism is that the agitation against the Council and against the Act has been engineered for political motives and chiefly fomented by the party Press. This again is an entire misrepresentation of the facts, invented by certain politicians and newspapers for party purposes.

Hard upon this, one reads that there are now two clearly defined and contending policies before the profession: one of "no service whatsoever," and one of "no service without the six points and adequate remuneration assured by the Commissioners." The former policy is sneered at as obviously dangerous; the latter belauded. But the very essence of the whole question is begged by the way in which the *Practitioner's* policy is misstated. It is not "no service whatsoever," but "no service without an amending Act" guaranteeing the six points which the Council of the British Medical Association were instructed to secure and could have secured, but did not. The difference between the two policies is thus seen to be merely whether Parliament or the Insurance Commissioners shall settle the future of the medical profession. It is true that the Commissioners cannot bamboozle the doctors more craftily than the House of Commons, or rather the Chancellor, did; so that there may be some hope from them. But surely the safer and more dignified way is to go back to Parliament and say "You tricked and intrigued with our representatives; now kindly revise your Act or we will have nothing to do with it."

But to return to the arguments of the fifty. They assure the profession that refusal to form panels will not "hang up" the medical benefits of the Act. They point out that the Commissioners have power to suspend medical benefits by giving the insured the equivalent in cash. So they have; but it is quite certain that they could not use that power, or at least that the Act would be absolutely damned and destroyed if they did. That clause was doubtless intended to frighten the profession, but it is a turnip-and-candle device whose hollowness could scarcely be more obvious. Still, going on the assumption that the clause would be put in action, the manifesto concludes that the result would be the destruction of the newly won unity of the profession. Yet it is this very unity which the manifestants are setting out themselves to destroy!

The alternative suggested is the continuation by the British Medical Association of those negotiations whose total failure in the hands of the present Council has been the cause of all the trouble. It is pleasant to be able to agree with some of the statements of the manifesto; thus it is admitted that the present provision for remunerating an efficient medical service is inadequate, and that thereby the scheme is fundamentally defective. Do then those who have issued this pamphlet imagine that the Commissioners can make money out of nothing? Large as the powers of the Commissioners are, they have only the disposal of certain funds allotted them by the Act, and those funds are calculated on an actuarial basis which allows only of " inadequate " medical remuneration. No; Parliament only can vary the financial provisions of this Act. Parliament endeavoured to put a heavy yoke upon the medical profession; and it is Parliament which will have to lift it off again. That is certain, *provided the medical profession stands firm.* The representative meeting summoned by the British Medical Association is proceeding in the Guildhall as we go to press. Judging by the trend of the proceedings so far, the representatives are not in the temper to be baulked of their opportunity of securing a profound change in the feeble policy of the Council of the Association. A decisive snub—practically a vote of no confidence—was administered to the latter at the outset over the constitution of the State Sickness Committee; and it will be surprising if other plain indications of the feeling of the medical profession are not forthcoming before the representatives disperse.

